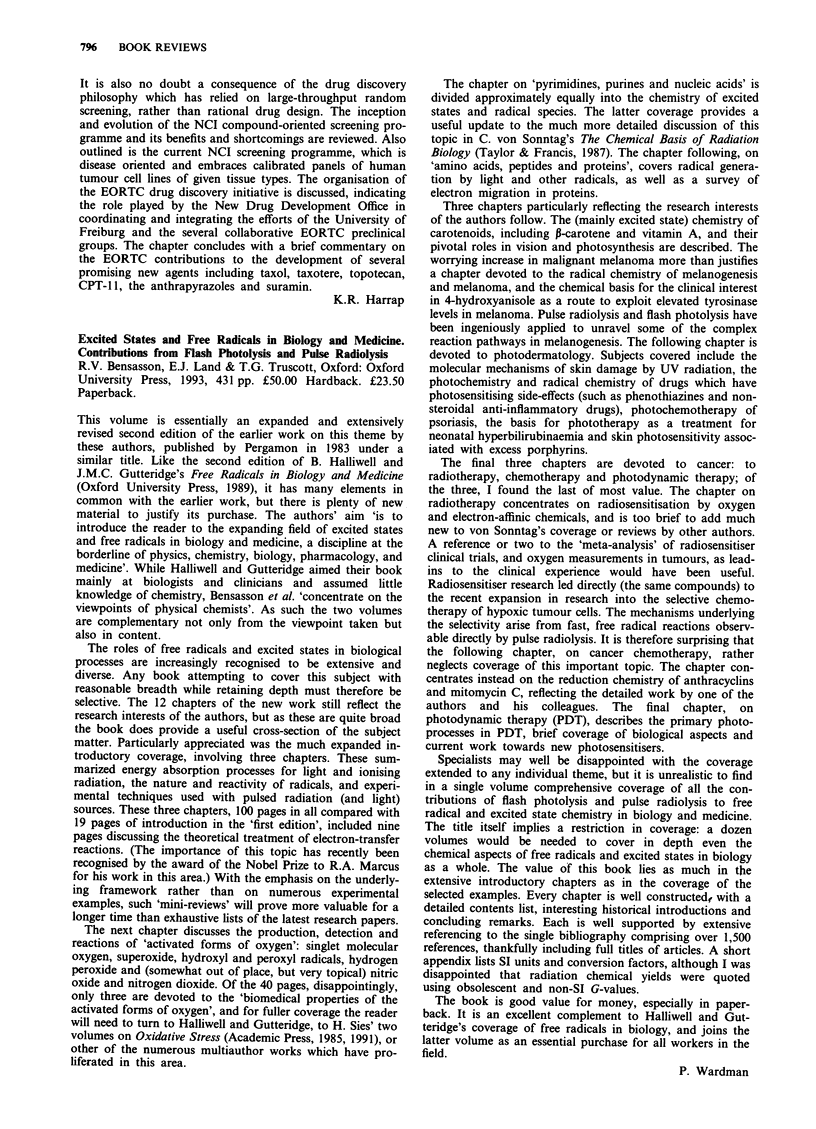# Excited States and Free Radicals in Biology and Medicine. Contributions from Flash Photolysis and Pulse Radiolysis

**Published:** 1994-04

**Authors:** P. Wardman


**Excited States and Free Radicals in Biology and Medicine. Contributions from Flash Photolysis and Pulse Radiolysis**
R.V. Bensasson, E.J. Land & T.G. Truscott, Oxford: Oxford University Press, 1993, 431 pp. £50.00 Hardback. £23.50 Paperback.

This volume is essentially an expanded and extensively revised second edition of the earlier work on this theme by these authors, published by Pergamon in 1983 under a similar title. Like the second edition of B. Halliwell and J.M.C. Gutteridge's *Free Radicals in Biology and Medicine* (Oxford University Press, 1989), it has many elements in common with the earlier work, but there is plenty of new material to justify its purchase. The authors' aim 'is to introduce the reader to the expanding field of excited states and free radicals in biology and medicine, a discipline at the borderline of physics, chemistry, biology, pharmacology, and medicine'. While Halliwell and Gutteridge aimed their book mainly at biologists and clinicians and assumed little knowledge of chemistry, Bensasson *et al.* 'concentrate on the viewpoints of physical chemists'. As such the two volumes are complementary not only from the viewpoint taken but also in content.

The roles of free radicals and excited states in biological processes are increasingly recognised to be extensive and diverse. Any book attempting to cover this subject with reasonable breadth while retaining depth must therefore be selective. The 12 chapters of the new work still reflect the research interests of the authors, but as these are quite broad the book does provide a useful cross-section of the subject matter. Particularly appreciated was the much expanded introductory coverage, involving three chapters. These summarized energy absorption processes for light and ionising radiation, the nature and reactivity of radicals, and experimental techniques used with pulsed radiation (and light) sources. These three chapters, 100 pages in all compared with 19 pages of introduction in the 'first edition', included nine pages discussing the theoretical treatment of electron-transfer reactions. (The importance of this topic has recently been recognised by the award of the Nobel Prize to R.A. Marcus for his work in this area.) With the emphasis on the underlying framework rather than on numerous experimental examples, such 'mini-reviews' will prove more valuable for a longer time than exhaustive lists of the latest research papers.

The next chapter discusses the production, detection and reactions of 'activated forms of oxygen': singlet molecular oxygen, superoxide, hydroxyl and peroxyl radicals, hydrogen peroxide and (somewhat out of place, but very topical) nitric oxide and nitrogen dioxide. Of the 40 pages, disappointingly, only three are devoted to the 'biomedical properties of the activated forms of oxygen', and for fuller coverage the reader will need to turn to Halliwell and Gutteridge, to H. Sies' two volumes on *Oxidative Stress* (Academic Press, 1985, 1991), or other of the numerous multiauthor works which have proliferated in this area.

The chapter on 'pyrimidines, purines and nucleic acids' is divided approximately equally into the chemistry of excited states and radical species. The latter coverage provides a useful update to the much more detailed discussion of this topic in C. von Sonntag's *The Chemical Basis of Radiation Biology* (Taylor & Francis, 1987). The chapter following, on 'amino acids, peptides and proteins', covers radical generation by light and other radicals, as well as a survey of electron migration in proteins.

Three chapters particularly reflecting the research interests of the authors follow. The (mainly excited state) chemistry of carotenoids, including β-carotene and vitamin A, and their pivotal roles in vision and photosynthesis are described. The worrying increase in malignant melanoma more than justifies a chapter devoted to the radical chemistry of melanogenesis and melanoma, and the chemical basis for the clinical interest in 4-hydroxyanisole as a route to exploit elevated tyrosinase levels in melanoma. Pulse radiolysis and flash photolysis have been ingeniously applied to unravel some of the complex reaction pathways in melanogenesis. The following chapter is devoted to photodermatology. Subjects covered include the molecular mechanisms of skin damage by UV radiation, the photochemistry and radical chemistry of drugs which have photosensitising side-effects (such as phenothiazines and non-steroidal anti-inflammatory drugs), photochemotherapy of psoriasis, the basis for phototherapy as a treatment for neonatal hyperbilirubinaemia and skin photosensitivity associated with excess porphyrins.

The final three chapters are devoted to cancer: to radiotherapy, chemotherapy and photodynamic therapy; of the three, I found the last of most value. The chapter on radiotherapy concentrates on radiosensitisation by oxygen and electron-affinic chemicals, and is too brief to add much new to von Sonntag's coverage or reviews by other authors. A reference or two to the 'meta-analysis' of radiosensitiser clinical trials, and oxygen measurements in tumours, as lead-ins to the clinical experience would have been useful. Radiosensitiser research led directly (the same compounds) to the recent expansion in research into the selective chemotherapy of hypoxic tumour cells. The mechanisms underlying the selectivity arise from fast, free radical reactions observable directly by pulse radiolysis. It is therefore surprising that the following chapter, on cancer chemotherapy, rather neglects coverage of this important topic. The chapter concentrates instead on the reduction chemistry of anthracyclins and mitomycin C, reflecting the detailed work by one of the authors and his colleagues. The final chapter, on photodynamic therapy (PDT), describes the primary photoprocesses in PDT, brief coverage of biological aspects and current work towards new photosensitisers.

Specialists may well be disappointed with the coverage extended to any individual theme, but it is unrealistic to find in a single volume comprehensive coverage of all the contributions of flash photolysis and pulse radiolysis to free radical and excited state chemistry in biology and medicine. The title itself implies a restriction in coverage: a dozen volumes would be needed to cover in depth even the chemical aspects of free radicals and excited states in biology as a whole. The value of this book lies as much in the extensive introductory chapters as in the coverage of the selected examples. Every chapter is well constructed, with a detailed contents list, interesting historical introductions and concluding remarks. Each is well supported by extensive referencing to the single bibliography comprising over 1,500 references, thankfully including full titles of articles. A short appendix lists SI units and conversion factors, although I was disappointed that radiation chemical yields were quoted using obsolescent and non-SI *G*-values.

The book is good value for money, especially in paperback. It is an excellent complement to Halliwell and Gutteridge's coverage of free radicals in biology, and joins the latter volume as an essential purchase for all workers in the field.

P. Wardman